# Type IV collagen α6 chain is a regulator of keratin 10 in keratinization of oral mucosal epithelium

**DOI:** 10.1038/s41598-018-21000-0

**Published:** 2018-02-08

**Authors:** Taishi Komori, Mitsuaki Ono, Emilio Satoshi Hara, Junji Ueda, Ha Thi Thu Nguyen, Ha Thi Nguyen, Tomoko Yonezawa, Takahiro Maeba, Aya Kimura-Ono, Takeshi Takarada, Ryusuke Momota, Kenji Maekawa, Takuo Kuboki, Toshitaka Oohashi

**Affiliations:** 1 Department of Oral Rehabilitation and Regenerative Medicine, Okayama University Graduate School of Medicine, Dentistry and Pharmaceutical Sciences, Okayama, 700-8525 Japan; 2 Department of Molecular Biology and Biochemistry, Okayama University Graduate School of Medicine, Dentistry and Pharmaceutical Sciences, Okayama, 700-8525 Japan; 3 Department of Biomaterials, Okayama University Graduate School of Medicine, Dentistry and Pharmaceutical Sciences, Okayama, 700-8525 Japan; 4 Department of Regenerative Science, Okayama University Graduate School of Medicine, Dentistry and Pharmaceutical Sciences, Okayama, 700-8525 Japan; 5 Department of Human Morphology, Okayama University Graduate School of Medicine, Dentistry and Pharmaceutical Sciences, Okayama, 700-8525 Japan

## Abstract

Keratinized mucosa is of fundamental importance to maintain healthy gingival tissue, and understanding the mechanisms of oral mucosa keratinization is crucial to successfully manage healthy gingiva. Previous studies have shown a strong involvement of the basement membrane in the proliferation and differentiation of epithelial cells. Therefore, first, to identify the keratinized mucosa-specific basement membrane components, immunohistochemical analysis for the six alpha chains of type IV collagen was performed in 8-week-old mice. No difference in the expression pattern of type IV collagen α1(IV) and α2(IV) chains was observed in the keratinized and non-keratinized mucosa. Interestingly, however, type IV collagen α5(IV) and α6(IV) chains specifically were strongly detected in the keratinized mucosa. To analyze the functional roles of the type IV collagen isoform α6(IV) in oral mucosa keratinization, we analyzed *Col4a*6*-*knockout mice. Epithelial developmental delay and low levels of KRT10 were observed in new-born *Col4a*6-knockout mice. Additionally, *in vitro* experiments with loss-of function analysis using human gingival epithelial cells confirmed the important role of α6(IV) chain in epithelial keratinization. These findings indicate that α112:α556 (IV) network, which is the only network that includes the α6(IV) chain, is one regulator of KRT10 expression in keratinization of oral mucosal epithelium.

## Introduction

Epithelial keratinization is of fundamental importance for protection against pathogens and mechanical stress, and is known to be regulated by direct (e.g., cell-to-cell contact) and indirect (e.g., paracrine activity of growth factors) interactions between epithelial cells, epithelial-mesenchymal cells and cell-basement membrane^1–4^.

In the oral cavity, keratinized mucosa is found in the gingiva and palate mucosa, whereas the non-keratinized mucosa is found in the buccal mucosa. Keratinized gingiva around the tooth and dental implant is critical to maintain a healthy condition of periodontal and peri-implant tissues, and to facilitate oral hygiene, as an insufficient volume of keratinized gingiva results in gingival inflammation^5–8^. However, following severe periodontal disease, tooth extraction and long-standing edentulism, loss of alveolar bone height is accompanied by a decrease in the amount and thickness of the keratinized gingiva, which has a very poor self-healing ability. Although free gingival graft is currently the gold standard for increasing the area of keratinized gingiva around both the natural tooth and dental implants, major disadvantages are related to the limited options of donor site, and post-operative complications^9^. Therefore, the understanding of the mechanisms behind the process of gingiva keratinization is essential for the development of novel techniques and materials for keratinized gingival tissue reconstruction and regeneration. However, the mechanisms regulating keratinization of gingiva still remain unclear.

Basement membrane separates the epithelial and mesenchymal tissues and it plays important roles in the determination of cell polarity, proliferation and differentiation^10–13^. In this study, we hypothesized that the basement membrane is a critical regulator of keratinization of the oral mucosal epithelium. The basement membrane, consisting of the lamina lucida and lamina densa, is composed of four major components (i.e., type IV collagen, laminin, nidogen, and perlecan), and separates the epithelium, mesothelium and endothelium from the connective tissue^10^. Type IV collagen is identified primarily in the skin within the basement membrane, and comprises six collagen chains (i.e., α1 to α6). These helical polypeptide α-chains form triple-helical protomers [α1α1α2 (α112), α3α4α5 (α345) and α5α5α6 (α556)], which further assemble into three major networks (α112:α112, α112:α556, α345:α345), interconnected by NC1 domain^14^. Type IV collagen α1 and α2 chains are expressed ubiquitously in basement membranes, although type IV collagen α3-α6 chains have a tissue specific distribution. Indeed, type IV collagen α1 to α5 chains, except α6 chain, are expressed in the kidney, whereas type IV collagen α1, α2, α5 and α6 chains, excluding α3 and α4 chains, are expressed in the skin^15^. It is well known that mutations in type IV collagen α3 to α5 chains causes Alport’s syndrome associated with glomerulonephritis, sensorineural deafness and eye abnormalities.

From these reports, we hypothesized that the difference in keratinization of palatal and buccal mucosa could be associated with the composition of the basement membrane. We herein focused especially on type IV collagen, and found that α5(IV) and α6(IV) chains were highly expressed in keratinized oral mucosa. Loss-of-function analysis using *Col4a6* knock out mice (*Col4a6*-KO mice) and gene expression knockdown with siRNA indicated that α112:α556 (IV) network, which is the only network that includes the α6(IV) chain, plays an important role in the keratinization of oral mucosa epithelial cells.

## Results

### Histological and morphological differences between palatal and buccal mucosa

**Figure 1:**
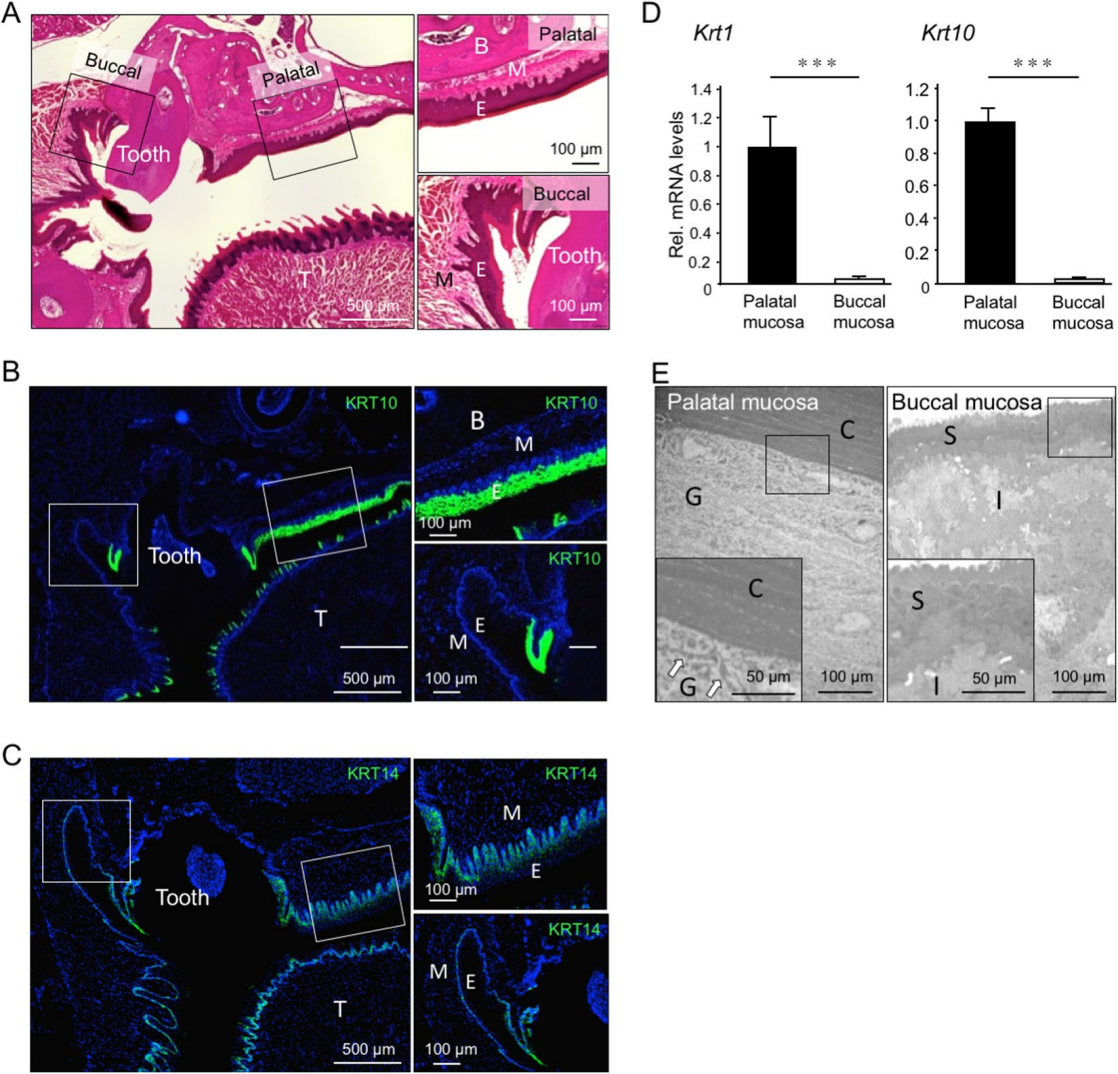
**C**omparison of palatal and buccal mucosa. Histological analysis of oral mucosa in 8-weeks-old mice. HE staining (**A**) and IHC staining for KRT10 (green) (**B**) or KRT14 (**C**) was performed using coronal sections of mouse head. Nuclei were counterstained with DAPI (blue). Boxes indicate the area shown at higher magnification in the right panels. E, epithelial tissue; M, Mesenchymal tissue; T, Tongue; B, bone. Note that KRT10 is highly expressed in keratinized mucosa, whereas KRT14 is expressed in basal cells of both keratinized and non-keratinized mucosa. (**D**) mRNA expression levels of *Krt1* and *Krt10* in palatal and buccal mocosa was measured by real time RT-PCR. The expression of each gene was normalized to that of *S29* ribosomal RNA. Bars represent the mean values and standard deviation (+/−SD) (n = 3). ***p < 0.001 (Student’s t-tests). Results are representative data of at least three independent experiments. (**E**) TEM image of palatal mucosa (left) and buccal mucosa (right). Boxes indicate the area shown at higher magnification. Arrows indicate keratohyalin granules. C, stratum corneum; G, Granular layer; S, Superficial layer; I, Intermediate layer.

First, to demonstrate that palatal and buccal mucosa in mice are distinct types of mucosa, histological and morphological analysis were performed. Histological findings showed the presence of parakeratosis in the external layer of both palatal and buccal mucosa (Fig. 1A). Keratin 1 (KRT1) and keratin 10 (KRT10), which are the major markers for keratinized mucosa, were highly expressed in the palatal mucosa, as shown by real time RT-PCR analysis (Fig. 1D). Further immunohistochemical (IHC) analysis confirmed the presence of KRT10 only in the palatal mucosa (Fig. 1B). On the other hand, KRT14 was detected in both keratinized and non-keratinized mucosa (Fig. 1C). Transmission electron microscopy (TEM) was also used to observe the details of palatal and buccal mucosa morphology. TEM images showed that palatal mucosa presented a stratum corneum and a granular layer, where keratohyalin granules were observed. On the other hand, in the buccal mucosa, these two layers were absent (Fig. 1E).

### Analysis of type IV collagen expression in basement membrane of keratinized and non-keratinized mucosa

**Figure 2:**
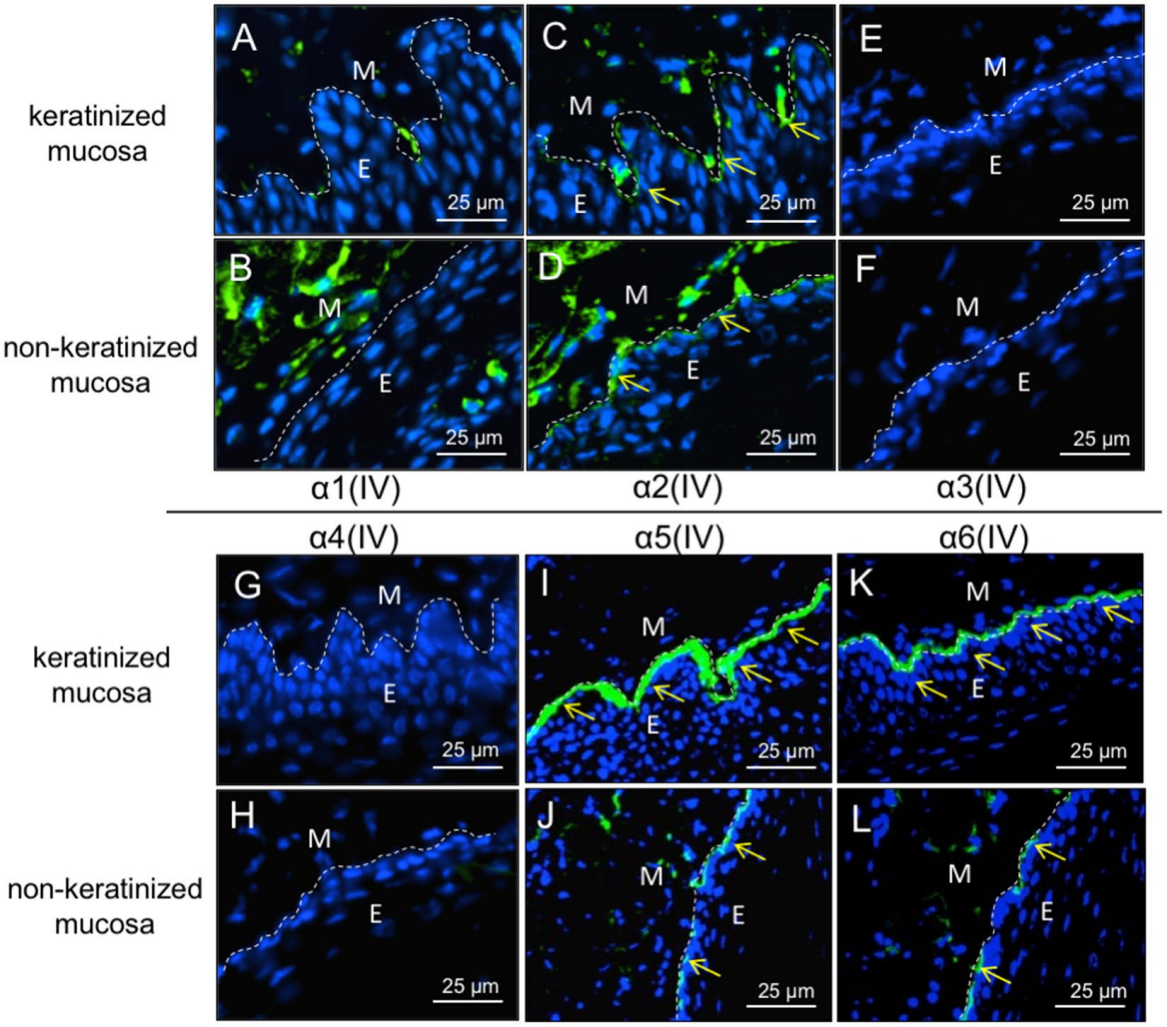
Analysis of the six alpha chains of type IV collagen in the basement membrane of keratinized and non-keratinized mucosa. IHC staining for α1(IV) (**A,B**), α2(IV) (**C,D**), α3(IV) (**E,F**), α4(IV) (**G,H**), α5(IV) (**I,J**), α6(IV) (**K,L**) was performed to compare the expression of these proteins in keratinized (**A**,**C**,**E**,**G**,**I**,**K**) and non-keratinized mucosa (**B**,**D**,**F**,**H**,**J**,**L**). Nuclei were counterstained with DAPI (blue). Yellow arrows indicate positive signals for each antibody in the basement membrane. Note that α5(IV) and α6(IV) chains are highly expressed in keratinized mucosa. Results are representative of at least three independent experiments. E, epithelial tissue; M, Mesenchymal tissue.

Previous reports have demonstrated that type IV collagen α1 and α2 chains express ubiquitously in basement membranes, although α3-α6 chains have a tissue specific distribution^16^. To characterize the differences in the composition of the basement membrane between keratinized and non-keratinized mucosa, we performed IHC analysis for the six α-chains of type IV collagen, and found no differences in α1(IV) and α2(IV) expression levels in both keratinized and non-keratinized mucosa (Fig. 2A–D). The α3(IV) and α4(IV) chains could not be detected in either mucosa (Fig. 2E–H), but could be detected in the kidney (Supplemental Fig. 1). Interestingly, we found that the protein levels of α5(IV) and α6(IV) chains in keratinized mucosa were markedly high, compared to non-keratinized mucosa in 8-week-old mice (Fig. 2I–L). Based on these results and on the fact that type IV collagen consists of the three distinct triple-helical networks (i.e., α112:α112, α112:α556, α345:α345), we hypothesized that the α112:α556 network of type IV collagen could play an important role in keratinization of oral mucosa. Since type IV collagen α6 chain is included only in the α112:α556 network, we performed a deeper analysis on the role of type IV collagen α6 chain in epithelial keratinization.

### Expression pattern of type IV collagen α6 chain and keratin 10 during embryonic development

**Figure 3:**
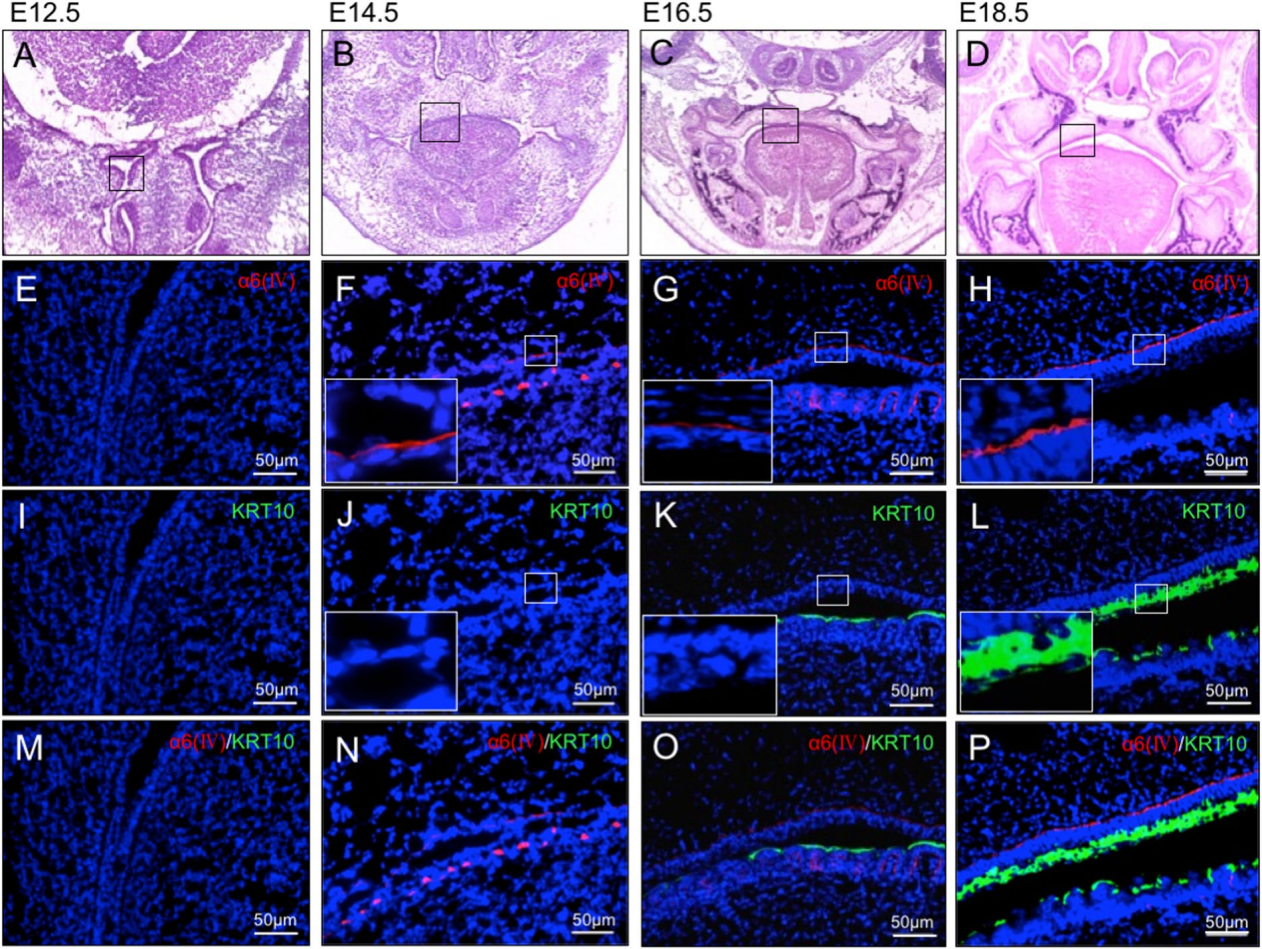
Expression analysis of α6(IV) and KRT10 during mouse embryonic development. The mucosal samples of mice embryos at E12.5 (**A**,**E**,**I**,**M**), E14.5 (**B**,**F**,**J**,**N**), E16.5 (**C**,**G**,**K**,**O**) and E18.5 (**D**,**H**,**L**,**P**) were collected. HE staining of the keratinized (palatal) mucosa is shown in **A**–**D**. Boxes indicate the area shown at higher magnification in the low panels. (**E**–**L**) show single staining for α6(IV) (red) and KRT10 (green) in palatal mucosa, and (**M**–**P**) are merged images. Nuclei were counterstained with DAPI (blue).

Secondary palatal development begins at embryonic day 12.5 (E12.5), and the palatal shelves, which have been vertically oriented on either side of the tongue, are elevated to the horizontal position at E14.5. Fusion between the two palatal shelves occurs by E15.5^17^. Therefore, in order to understand the chronological order of α6(IV) and KRT10 expression during palatal development, HE staining and IHC staining were performed using E12.5 to E18.5 mice including the developmental stages before, during and after palate formation and palatal mucosa keratinization (Fig. 3A–D). As shown in Fig. 3E–H, the protein expression levels of α6(IV) could be detected in palatal mucosa onward E14.5. Interestingly, however, KRT10 could only be observed at E18.5 (Fig. 3I–P). Therefore, these results suggested that the α556(IV) protomer contained in α112-α556 network could be an important factor involved in keratin formation in keratinized mucosa.

### Functional analysis of type IV collagen using *Col4a6-knockout* mice

**Figure 4:**
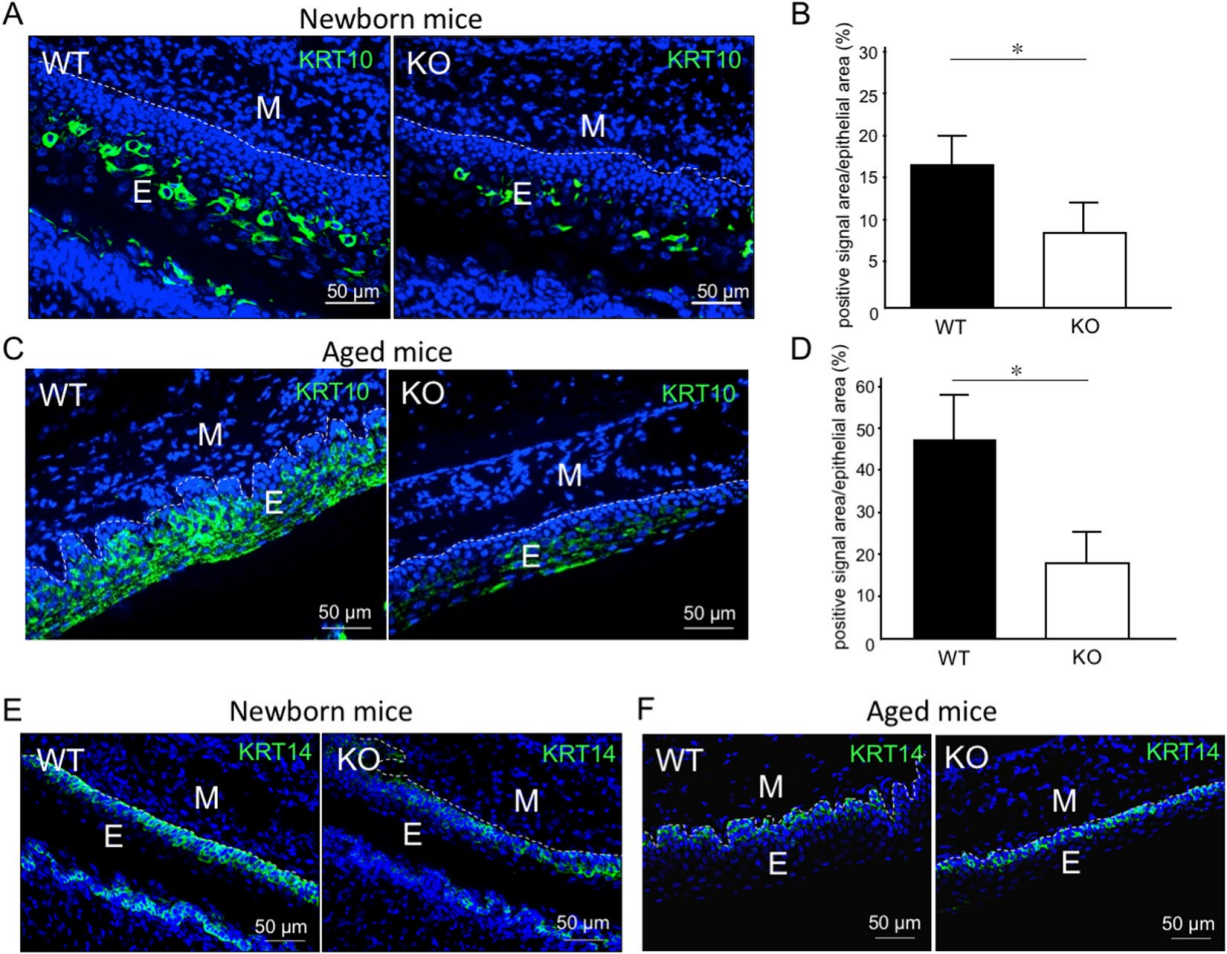
Comparison of keratinized mucosa between new-born and aged WT and *Col4a6*-KO mice. (**A**,**C**) IHC staining for KRT10 (green) in palatal mucosa of WT and *Col4a6*-KO mice in new-born (**A**) and 28-week-old aged mice (**D**). Nuclei were counterstained with DAPI (blue). (**E**) Epithelial tissue; M, Mesenchymal tissue. The percentage of positive fluorescent signal for KRT10 in the area of palatal mucosa of new-born and aged mice is shown in graphs B and D, respectively. Bars represent the mean values and standard deviation (+/−SD) (n = 3). *p < 0.05 (Student’s t-tests). (**E**–**F**) IHC staining for KRT14 (green) in palatal mucosa of WT and *Col4a6*-KO mice in new-born (**E**) and 28-week-old aged mice (**F**). Note that KRT14 levels are higher in newborn WT mice compared to *Col4a6*-KO mice. In adult mice, the levels of KRT14 are identical in both WT and *Col4a6*-KO mice. Results are representative of at least three independent experiments.

Next, we used type IV collagen knockout (*Col4a6-*KO) mice to investigate the role of type IV collagen α6 chain on keratinization of oral mucosa. Immunohistological analysis showed a low level of KRT10 (Fig. 4A,B) and KRT1 (Supplemental Fig. 2A) in new-born *Col4a6*-KO mice. Of note, there was no notable difference in the expression levels of perlecan between *Col4a6*-KO and WT mice (Supplemental Fig. 3). Since aging has been related to a decrease in type IV collagen expression^18^, we analyzed the keratinized mucosa of 28-week-old aged mice. As shown in Fig. 4C,D, the expression levels of KRT10 were decreased in *Col4a6*-KO mice compared with WT mice.

It is well known that KRT5 and KRT14 are expressed in the basal cells of oral mucosa and involved in the formation of strong networks that help attach keratinocytes together. As these cells enter the terminal differentiation program, KRT5 and KRT14 are substituted by KRT1 and KRT10. Therefore, we also checked the expression level of KRT14, and found that it was decreased in new-born *Col4a6*-KO mice compared with WT mice (Fig. 4E), but the expression was identical in aged (28-week-old) WT and *Col4a6*-KO mice (Fig. 4F). Additional IHC analysis for α5(IV) in *Col4a6*-KO mice showed the loss of α5(IV) in keratinized mucosa (Supplemental Fig. 2B,C). From these results, we concluded that α556(IV) protomer is absent, and that α112:α556 network is not synthesized in keratinized mucosa of *Col4a6*-KO mice.

### Functional analysis of type IV collagen α6 *in vitro*

**Figure 5:**
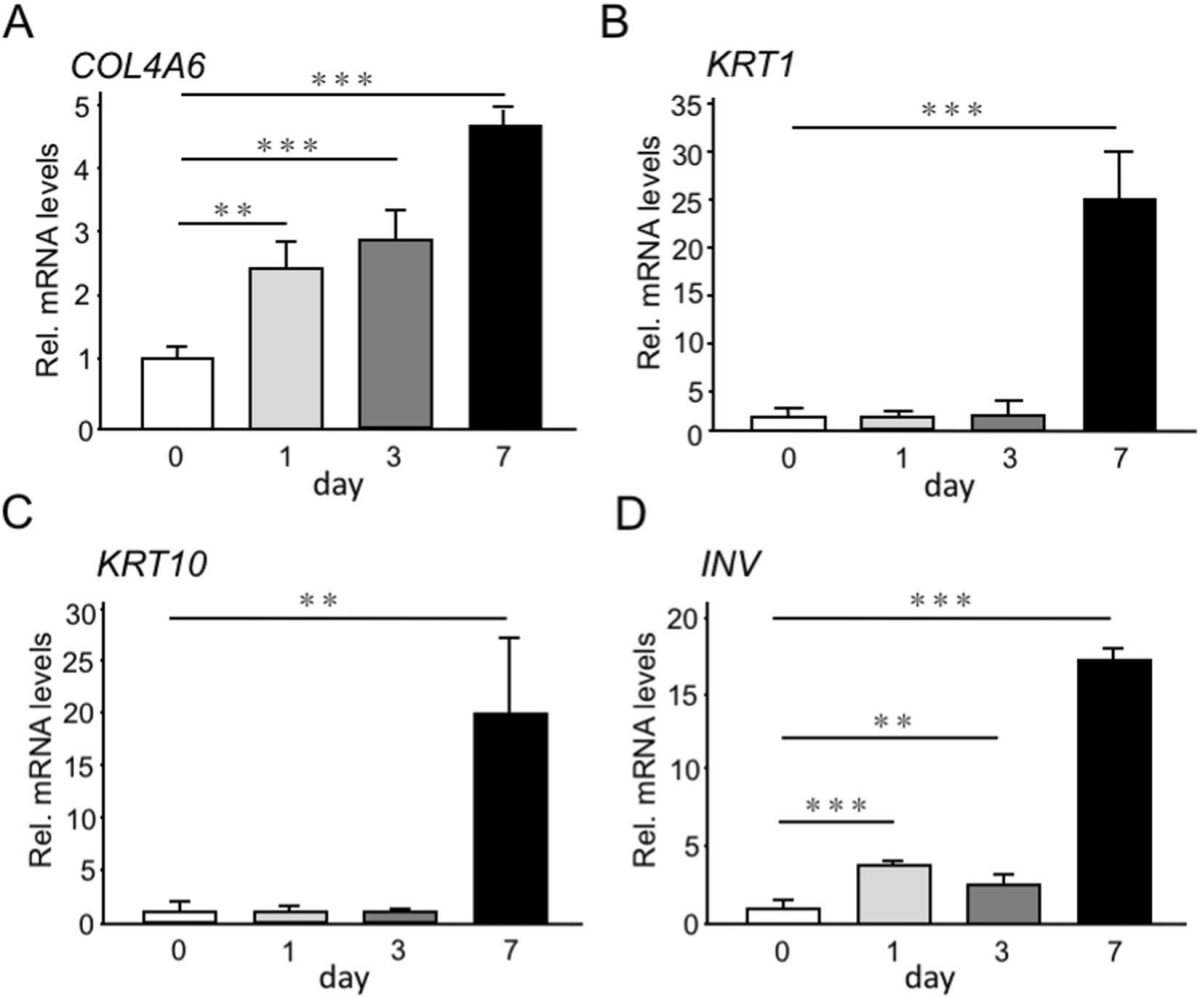
Expression analysis of *COL4A6* and keratin markers during epithelial keratinization *in vitro*. hGECs were seeded in the ThinCert cell culture inserts (3D culture), and total RNA was collected after 0, 1, 3 and 7 days. mRNA expression levels of *COL4A6* (**A**), *KRT1* (**B**), *KRT10* (**C**) and *INV* (**D**) were measured by real time RT-PCR. The expression of each gene was normalized to that of *S29* ribosomal RNA. Bars represent the mean values and standard deviation (+/−SD) (n = 3). **p < 0.01, ***p < 0.001 (ANOVA, Tukey multiple comparison test).

A previous study reported that only epithelial cells produce *Col4a6* in colon^19^; thus, to obtain a deeper insight into the effect of type IV collagen α6 chain on epithelial keratinization, we cultured hGECs using 3D culture methods to induce epithelial keratinization, and analyzed the gene expression pattern of *COL4A6* and keratinization markers by real time RT-PCR. Gene expression levels of *COL4A6* increased by 2.4 times after 1 day and 4.7 times after 7 days (Fig. 5A). Strikingly, the gene expression levels of *KRT1*, *KRT10* and involucrin *(INV)*, one of keratinization-related genes, were markedly low in the initial days of culture, but increased dramatically at day 7 (Fig. 5B–D). Accordingly, the gene expression levels of *KRT5, KRT13, KRT14, KRT15* and *KRT16*, which have been identified to be expressed in oral mucosa, increased markedly after 7 days of culture (Supplemental Fig. 4). Of note, when hGESs were cultured in normal culture dish (2D culture model), *KRT1* mRNA expression could not be detected and *KRT10* expression level was low and did not increase even after 28 days in 2D culture (Supplemental Fig. 5); indicating that the 3D culture system is required for proper mucosa keratinization.

**Figure 6:**
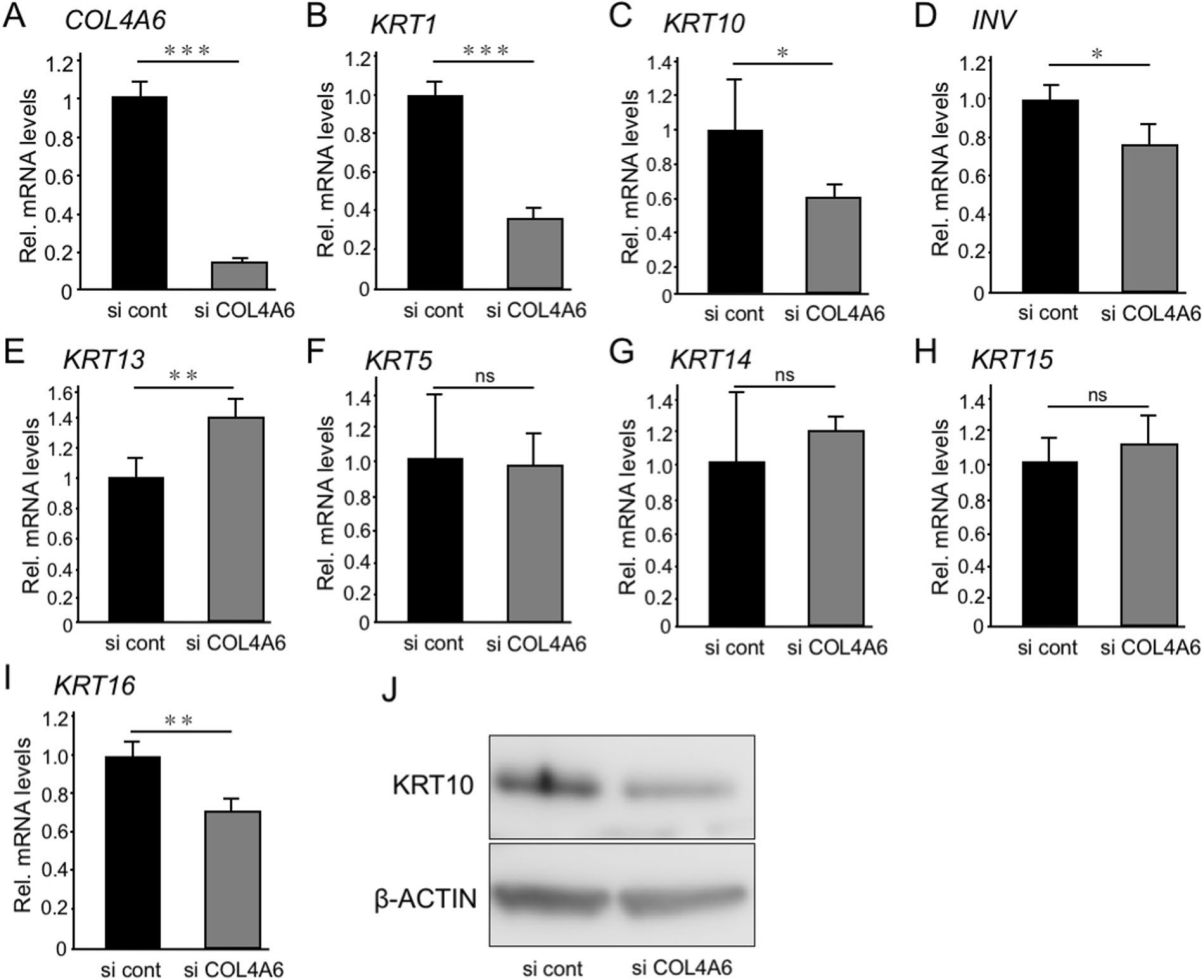
Functional analysis of *COL4A6* knockdown *in vitro*. hGECs transfected with siRNA targeting *COL4A6* gene were seeded in the ThinCert cell culture inserts (3D culture), and total RNA and cell lysates were collected after 3 days. mRNA expression levels of *COL4A6* (**A**), *KRT1* (**B**), *KRT10* (**C**), *INV* (**D**), *KRT13* (**E**), *KRT5* (**F**), *KRT14* (**G**), *KRT15* (**H**) and *KRT16* (**I**) were measured by real time RT-PCR. The expression of each gene was normalized to that of *S29* ribosomal RNA. Bars represent the mean values and standard deviation (+/−SD) (n = 3). *p < 0.05, **p < 0.01, ***p < 0.001, ns, no significant difference (Student’s t-tests). Western blot analysis for KRT10 is shown in (**J**). Full length of western blot data is shown in Supplemental Fig. 7. Results are representative of at least three independent experiments.

Next, we investigated the effect of type IV collagen α6 chain knockdown by inhibition of *COL4A6* expression with siRNA specific for *COL4A6* on epithelial keratinization using the 3D culture model. Confirmation experiments showed that the expression of *COL4A6* mRNA (Fig. 6A), but not that of *COL4A1, COL4A2 or COL4A5* (Supplemental Fig. 6), was significantly decreased by *COL4A6 siRNA* in hGECs. As shown in (Fig. 6B–D,I), mRNA levels of *KRT1*, *KRT10*, *INV* and *KRT1*6 (marker of keratinocyte hyper-proliferation) decreased upon inhibition of *COL4A*6 expression. The decrease in KRT10 protein levels was also confirmed by western blot (Fig. 6J). On the other hand, blockade of *COL4A*6 in hGECs induced an increase in gene expression levels of *KRT13*, a marker of non-keratinized epithelia, but induced no significant changes in the gene expression levels of *KRT5*, *KRT14* and *KRT15*, which are expressed in basal cells in oral mucosa (Fig. 6E–H). Collectively, these results demonstrate that α556(IV) protomer contained in α112-α556 network is one of regulator of oral mucosa keratinization.

## Discussion

In the oral cavity, keratinized and non-keratinized mucosa are consecutive tissues, despite the differences in their morphology and function. Keratinization of oral mucosa is important for the maintenance of healthy periodontal tissue and long-term prognosis of natural tooth and dental implants. However, the molecular mechanism behind keratinization of oral mucosal epithelium is still unclear. Mutations in type IV collagen have been reported to cause Alport’s syndrome associated with glomerulonephritis, sensorineural deafness^20,21^. Therefore, we hypothesized that the basement membrane components could be one important factor regulating keratinization in palatal mucosa. The basement membrane, which composition is tissue-specific and undergoes dynamic changes during development and aging, plays important biological function in the development and maintenance of tissues and organs^22^. In this study, we demonstrated that the expression of α6(IV) chain preceded the expression of KRT10 during palatal development (Fig. 3), as well as in *in vitro* 3D culture of hGECs (Fig. 6). These results indicate a novel role of α6(IV) as one of the regulators of keratinization of oral mucosal epithelium. Since, type IV collagen α1(IV) and α2(IV) chains were expressed equally in keratinized and non-keratinized mucosa, and α3(IV) and α4(IV) chains were not detected in either of the tissues, the results highlight the importance of type IV collagen α6 chain, especially the α122:α556 network, in mucosa keratinization.

The importance of other type IV collagen chains have already been demonstrated in embryogenesis and diseases. Deletion of both *Col4a1/Col4a2* locus in mice is known to cause growth retardation and embryonic lethality^12^. Mice deficient of α3(IV) or α5(IV) are viable, but develop renal failures resembling those of Alport’s syndrome^23–25^. However, ablation of α6(IV) does not disrupt basement membrane assembly. Additionally, no notable phenotype in the kidney was observed. Therefore, this study is the first to demonstrate a phenotype of *Col4a6* deletion in the keratinization of oral mucosa in mice.

However, the cellular origin of type IV collagen, from epithelial cells or mesenchymal cells, is still not well understood. It has been reported that the mRNA of *COL4A2* is observed in both epithelial and mesenchymal cells in colon, but the mRNA of *COL4A6* is localized in the epithelial cells^19^. In the present study, we could not show the localization of *Col4a*6 mRNA by using *in situ* hybridization. However, we could observe the increased *Col4a6* mRNA level during epithelial keratinization of hGECs in the 3D culture model, indicating, at least, that epithelial cells can synthesize COL4A6 in oral mucosa. From these data, we assume that epithelial cells are able to secrete α6(IV) chain, which in turn could activate intracellular pathways that direct cell differentiation in an autocrine manner. Additionally, the epithelial-mesenchymal interaction could play fundamental roles in determining the differential expression of α6(IV) chain in keratinized mucosa. In fact, we had performed a preliminary study using hGECs, which were co-cultured with mesenchymal cells isolated either from keratinized or non-keratinized mucosa. We observed that keratinization occurred only in hGECs co-cultured with mesenchymal cells from keratinized mucosa. Therefore, there may exist numerous factors activated by the epithelial-mesenchymal cell interaction that could induce the initial synthesis and secretion of α6(IV) chain in cells forming the keratinized mucosa.

It is conceivable from our IHC data that, collagen IV α112:α112 and α112:α556 networks exist in the basement membrane of both keratinized and non-keratinized gingiva, and a larger amount of α112:α556 network exists in keratinized gingiva. Interestingly, in the *Col4a6-*KO mice, not only α6(IV) chain, but also α5(IV) chain were absent in basement membrane of gingiva (Supplemental Fig. 2). It has been reported that the mutation of α5 chain in X-linked Alport’s syndrome caused the loss of α6 chain, and failure of assembly of α556 protomer and α112:α556 network (Zheng, A. J. Pathology, 1999). Accordingly, there could also have an absence of α112:α556 network due to loss of α6 chain in the keratinized gingiva of *Col4a6-*KO mice.

A previous study reported that as many as 10–12% of basal layer cells are stem cells in skin and colon^26^, and these stem cells play an important role in the development and maintenance of epithelium by regulating cell proliferation and differentiation^2^. Basement membrane, especially type IV collagen which serves as a ligand for integrin α1β1 and α2β1, is supposed to contribute to the niche of tissue-specific stem cells. It has been reported that integrin β1 positive keratinocytes have high properties of self-renewal, compared with integrin β1 negative cells^27^. Additionally, integrin β1 deletion in skin epithelial cells caused not only a disorder in skin wound healing^28^ but also severe defects in basement membrane assembly and organization in mice^29^. In oral mucosa, oral keratinocyte stem cells are located in basement membrane of epithelium^2^, and have high affinity to type IV collagen^30^. Based on the fact that the development of epithelium is delayed in *Col4a6*-KO new-born mice, and that cell proliferation marker (cyclin D1) was also decreased in *Col4a6* silenced hGECs (data not shown), it was estimated that type IV collagen, especially α6(IV) chain, may provide a niche environment for keratinocyte stem cells, and regulate keratinocyte proliferation and differentiation in oral mucosal epithelium. However, the biological mechanism of α6(IV) chain on stem cells has not been fully understood. Further researches, such as immunoassay and cell receptor assay using the recombinant protein α556(IV), are necessary and expected.

Other members of the basement membrane, such as laminin, nidogen and perlecan have also been reported to play important roles in determining epithelial cell fate^31–33^. Nevertheless, IHC analysis showed no difference in perlecan levels in keratinized gingiva and non-keratinized gingiva between new-born *Col4a6*-KO and WT mice. Due to the large number of molecules in the basement membrane, and possible compensation effects, further investigations are necessary to allow a deeper insight on the role of other components of the basement membrane in the keratinization of oral mucosal epithelium.

In summary, our study demonstrated that *α*5(IV) and *α*6(IV) chains were highly expressed in keratinized mucosa. Keratinization of oral mucosal epithelium of *Col4a6*-KO mice was decreased in new-born mice and aged mice. Additionally, down-regulation of *COL4A6* in hGECs caused suppression of epithelial keratinization. Collectively, these data indicate that type IV collagen *α*6 chain, and consequently α112:α556 network, is involved in KRT10 expression in keratinization of oral mucosal epithelium.

## Materials and Methods

### Animal experiments

Pregnant and 8-weeks-old c57BL/6 J mice were purchased from CLEA Japan (Tokyo, Japan). *Col4a6*-KO mice were generated by replacing parts of exon 2 and intron 2 with a Neomycin cassette in *Col4a6* gene as described before^34^. *Col4a6*-KO mice were then backcrossed with C57BL/6 J (Charles River) for ten generations. The animal experiment protocols used in this study (OKU-2016495, OKU-2017051) were approved by Okayama University Animal Research Committee. All animals were handled according to the guidelines of Okayama University Animal Research Committee.

### Histological analysis

For preparation of frozen sections from non-fixed and un-decalcified hard tissues, Kawamoto’s film methods were used^35,36^. Briefly, samples were freeze-embedded with super cryoembedding medium (SECTION-LAB Co. Ltd., Hiroshima, Japan) and cut in thickness of 5 μm with tungsten carbide blade after mounting the adhesive film onto the sample surface. Samples were then immediately fixed with 4% paraformaldehyde (PFA) for 20 min and stained with hematoxylin and eosin. IHC analysis for type IV collagen was performed according to the previous reports^37,38^. Briefly, the specimens were fixed with acetone for 20 minutes, followed by incubation with primary antibodies at 4 °C overnight, after blocking with 5% normal goat serum (Life technologies) containing 1% BSA (Sigma, St Louis, MO, USA) for 60 minutes at room temperature. Monoclonal primary antibodies specific for type IV collagen (α1 [H11], α2 [H22], α3 [H31], α4 [RH42], α5 [b14], and α6 [B66]) were used. These antibodies were kind gift from Dr. Sado (Okayama Univ., Japan). All antibodies for type IV collagen were diluted to 1:100 except for H22, which was diluted to 1:50. H11-, H22-, H31-, and b14-stained sections were treated with 6 M urea in 0.1 M glycine/HCl (pH 3.5) for 10 minutes and B66 for 1 min before blocking. Primary antibodies for KRT10 (ab76318), KRT1 (ab185629) and KRT14 (ab181595) were purchased from Abcam (Cambridge, UK). After washing, the specimens were incubated with secondary antibody Alexa Fluor 488 donkey anti-rabbit IgG (Life technologies, Tokyo, Japan), Alexa Fluor 488 donkey anti-rat IgG (Life technologies) or Alexa Fluor 647 anti-rabbit for 60 minutes at room temperature. All images were taken by fluorescence microscope. Quantification of KRT10 positive area in epithelium was based on fluorescent IHC images taken with BZ-700 microscope and analyzed with a BZ analyzer (Keyence, Osaka, Japan).

### Real time RT-PCR analysis

The collected palatal and buccal mucosa were homogenized using PowerMasher II (Nippi, Tokyo., Japan) and BioMasher II (Nippi, Tokyo., Japan). After homogenization, total RNA was extracted using TRIzol^®^ Reagent (Life technologies) according to a conventional method and purified using a PureLink^®^ RNA Mini Kit (Life technologies). The total RNA from cultured cells was extracted and purified using PureLink^®^ RNA Mini Kit.

RNA samples were reverse-transcribed by using iScript cDNA synthesis kit (Bio-Rad, Hercules, CA, USA). Real time RT-PCR was performed to quantify the expression of the target gene by using KAPA SYBR FAST qPCR Master Mix (KAPA BIOSYSTEMS, Wilmington, MA, USA) and CFX96 real-time system (Bio-Rad). The levels of mRNAs of interest were normalized to that of the reference gene S29. Primer sequences are shown in Table 1.

**Table 1:** The base sequence of the primer used for RT-PCR.

Target gene	Type	GeneBank registration number	Primer set
*S29*	human	BC032813	5′-TCTCGCTCTTGTCGTGTCTGTTC-3′(S)
			5′-ACACTGGCGGCACATATTGAGG-3′(AS)
*KRT1*	human	BC063697	5′-CTTACTCTACCTTGCTCCTACT-3′(S)
			5′-AAATCTCCCACCACCTCC-3′(AS)
*KRT5*	human	BC042132	5′-TGTTGTCACAAGCAGTGTT-3′ (S)
			5′-ACTGAGCCCACCACTTAG-3′(AS)
*KRT10*	human	NM_000421	5′-ACACCGCACAGAACCACCACTC-3′(S)
			5′-GGCAGGCACAGGTCTTGATGAAC-3′(AS)
*KRT13*	human	BC077718	5′-ACCTCTGTTACCACGACTT-3′(S)
			5′-GCCTACGGACATCAGAAGT-3′(AS)
*KRT14*	human	BC042437	5′-ACAGATCCCACTGGAAGAT-3′(S)
			5′-AGATAATGAAGCTGTATTGATTGC-3′(AS)
*KRT15*	human	BT007261	5′-TGCTGCTTGACATAAAGACA-3′(S)
			5′-CTACCACCACCTCCTGAA-3′(AS)
*KRT16*	human	AF061812	5′-TGAGATGGAGCAGCAGAG-3′(S)
			5′-GCGGGAAGAATAGGATTGG-3′(AS)
*INV*	human	BC046391	5′-CCTCAGATCGTCTCATACAAG-3′(S)
			5′-ACAGAGTCAAGTTCACAGATG-3′(AS)
*COL4A1*	human	NM_001845	5′-GGTGCCTTCCATACTGTTT-3′(S)
			5′-GCTACTGAGTCTGTAATTCCATT-3′(AS)
*COL4A2*	human	NM_001846	5′-GCAAAGCAGCAACTATTCAC-3′(S)
			5′-GCAGCATTCAAACTTCATACAA-3′(AS)
*COL4A5*	human	BC035387	5′-GACAAAGGTGATCCTGGTATT-3′(S)
			5′-CTGCTCAAGTATGTGCCTAA-3′(AS)
*COL4A6*	human	NM_001847	5′-TGATTTGGATGATTGTGTGACT-3′(S)
			5′-GACTGATTAGGCGATTAGGAAGA-3′(AS)
*S29*	mouse	NM_009093	5′-GGAGTCACCCACGGAAGTTCGG-3′(S)
			5′-GGAAGCACTGGCGGCACATG-3′(AS)
*Krt1*	mouse	BC117843	5′-ACATTTCAAAGAGGACTTCAGAT-3′(S)
			5′-AAAGACAAACTCGCAAACAC-3′(AS)
*Krt10*	mouse	NM_010660	5′-AGGACGATTATTGAGGAGGT-3′(S)
			5′-AAGTGTTTCTTGGTTTCTGATTC-3′(AS)

S: sense, AS: antisense.

### Morphological analysis

The palatal and buccal mucosa were harvested from eight-week-old female c57BL/6 J mice. The collected samples were fixed with 2% glutaraldehyde and 2% PFA overnight. After post-fixation with 1% osmium tetroxide and dehydration with ethanol, the samples were embedded in spurr low-viscosity embedding media (Polysciences, Warrington, PA, US). Ultrathin sections were then cut using a diamond knife and microtome (LEICA EM UC7, Leica Mikrosysteme GmbH, Vienna, Austria). The sections were double-stained with uranyl acetate and lead citrate and observed using a transmission electron microscope (TEM: H-7650, HITACHI, Tokyo, Japan).

### Cells and culture medium

Human gingival epithelial cells (hGECs) were purchased from CELLnTEC advanced cell systems AG (Stauffacherstrasse, Bern, Switzerland). hGECs were cultured in CnT-prime epithelial culture medium (CELLnTEC advanced cell systems AG, Stauffacherstrasse) at 37 °C in 5% CO_2_. To induce differentiation of hGECs, the cells were seeded onto cell culture inserts in a 12-well plate (ThinCert^TM^, Greiner Japan, Tokyo, Japan) using CnT-Prime 3D barrier medium (3D-medium, CELLnTEC advanced cell systems AG, Stauffacherstrasse). Briefly, the cells were seeded at 5.0 × 10^5^ cells in the upper chamber with 0.5 mL of 3D-medium. Additional 5 mL of 3D-medium was added to the lower chamber. For the down-regulation of the *Col4a6* gene, 5 nM of siRNA targeting the *Col4a6* gene (StelthTM SiCol4a6; Life Technologies) was transfected into hGECs using Lipofectamine RNAiMAX (Life Technologies), according to the manufacturer’s instructions. StelthTM RNAi Negative Control High GC Duplex (Life Technologies) was used as the negative control. Transfected hGECs were seeded at 2.0 × 10^6^ cells in the upper chamber with 0.5 mL of 3D-medium, and 5 mL of 3D-medium was added to the lower chamber. After 24 h, 3D-media of both chambers were aspirated, and 4 mL of 3D-medium was added only to the lower chamber, and the cells were cultured for additional 7 days.

### Western blot analysis

hGECs were lysed using M-PER (Mammalian Protein Extraction Reagent; Thermo, Waltham, Massachusetts, USA) supplemented with a Protease Inhibitor Cocktail (Roche, Indianapolis, IN, USA). Cell debris were removed from lysates by centrifugation at 12,000 rpm for 10 min at 4 °C. Protein concentration in the cell lysate was determined by using Pierce BCA Protein assay kit (Thermo). Twenty five micrograms of the total protein was separated in precast polyacrylamide gels (NuPage, Life Technologies) by electrophoresis and then transferred onto polyvinylidene fluoride membranes (PVDF; GE Healthcare Life sciences, Buckinghamshire, UK) at 30 V for 2 h. Blots were blocked and incubated with primary antibodies against KRT 10 (ab76318, 1:2000, Abcam) or β-actin (1:2000; Sigma), which was used as the loading control. Membranes were then washed, and incubated with goat anti-rabbit lgG-HRP (1:2000; Santa Cruz Biotechnology, Santa Cruz, CA, USA) or goat anti-mouse lgG-HRP (1:2000; Santa Cruz Biotechnology) for 1 h at room temperature. The blots were developed using Forte western HRP Substrate (Millipore), and visualized with Image Quant LAS 4000 mini (Fujifilm, Tokyo, Japan).

### Statistical analysis

The results obtained from quantitative experiments were reported as the mean values ± SD. Statistical analyses were performed with one-way factorial ANOVA followed by Tukey’s multiple comparison tests or Student’s unpaired t-tests.

## Electronic supplementary material

Supplementary Figure
