# Supplementary material for: Type IV collagen α6 chain is a regulator of keratin 10 in keratinization of oral mucosal epithelium
Source: Sci Rep. 2018 Feb 8;8:2612. doi: 10.1038/s41598-018-21000-0 (PMC5805778; doi:10.1038/s41598-018-21000-0)
Supplement: Supplementary file 1 — Supplementary Figure [file 41598_2018_21000_MOESM1_ESM.doc]

**Type IV collagen α6 chain is a regulator of keratin 10 in keratinization of oral mucosal epithelium**

Taishi KOMORI^1^, Mitsuaki ONO^2*^, Emilio Satoshi HARA^3^, Junji UEDA^1^, Ha Thi Thu NGUYEN^1^, Ha Thi NGUYEN ^1^, Tomoko YONEZAWA^2^, Takahiro MAEBA^2^, Aya ONO^1^, Takeshi TAKARADA^4^, Ryusuke MOMOTA^5^, Kenji MAEKAWA^1^, Takuo KUBOKI^1^, Toshitaka OOHASHI^2^

*^1^Department of Oral Rehabilitation and Regenerative Medicine, ^2^Department of Molecular Biology and Biochemistry, ^3^Department of Biomaterials, ^4^Department of Regenerative Science, ^5^Department of Human Morphology, Okayama University Graduate School of Medicine, Dentistry and Pharmaceutical Sciences*

**Supplemental Figure 1**

Immunohistochemistry for α3 (IV) (A) and α4 (IV) (B) (green) was performed using renal sections of 8-week-old mice. Nuclei were counterstained with DAPI (blue). Boxes indicate the area shown at higher magnification in the lower panels (C-D).


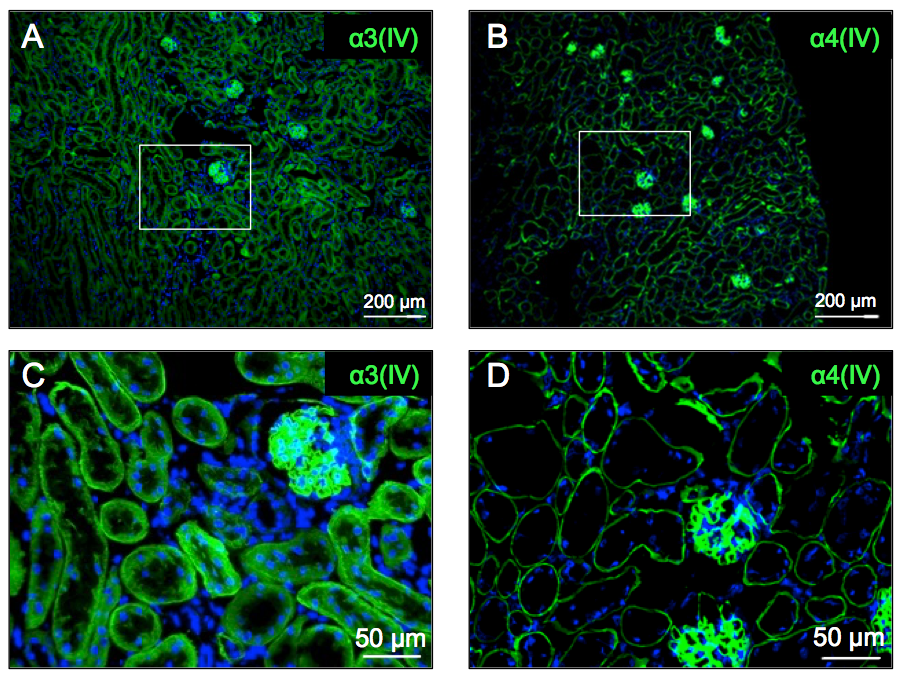


**Supplemental Figure 2**

Immunohistochemical staining for KRT1 (green) in palatal mucosa of WT and *Col4a6*-KO newborn mice (A) and for α5(IV) and α6(IV) (green) in palatal mucosa of WT and *Col4a6*-KO aged mice (B-C). Nuclei were counterstained with DAPI (blue). E, epithelial tissue; M, Mesenchymal tissue.


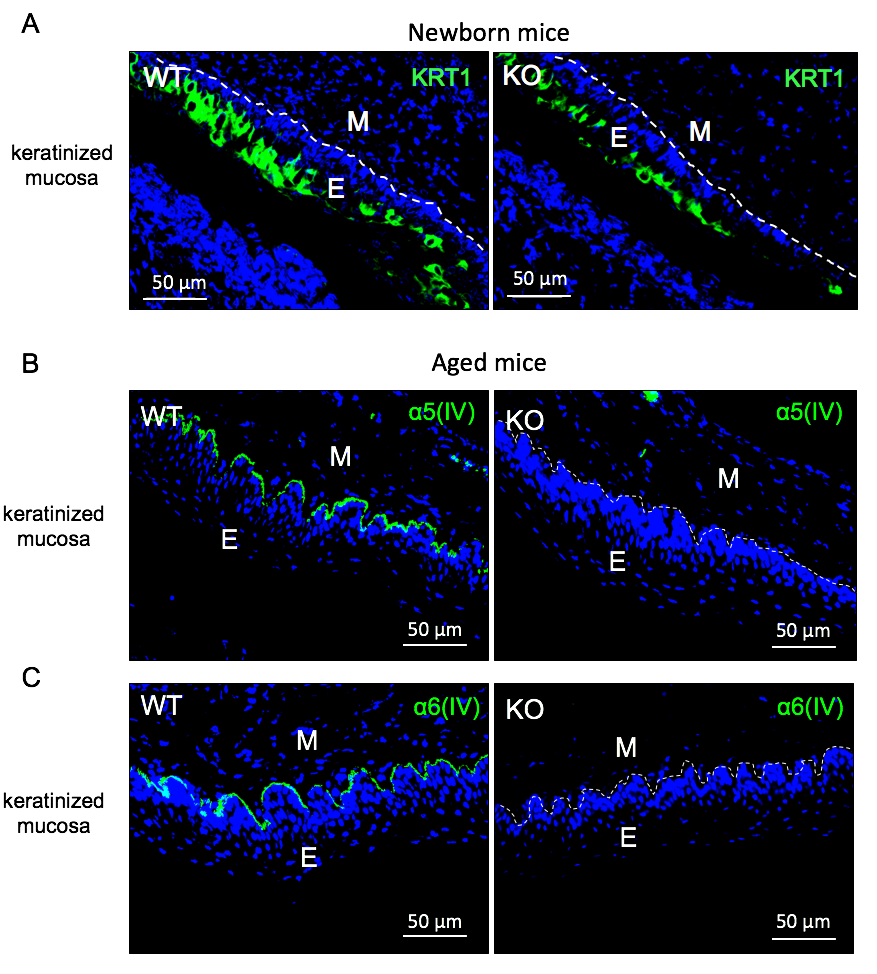


**Supplemental Figure 3**

Immunohistochemistry for perlecan (green) in keratinized and non-keratinized mucosa (A) in WT adult mice, and in new-born WT and *Col4a6*-KO mice (B). Nuclei were counterstained with DAPI (blue). Yellow arrows indicate positive area in basement membrane. Results are representative of at least three independent experiments. E, Epithelial tissue; M, Mesenchymal tissue. Results are representative data of at least three independent experiments.


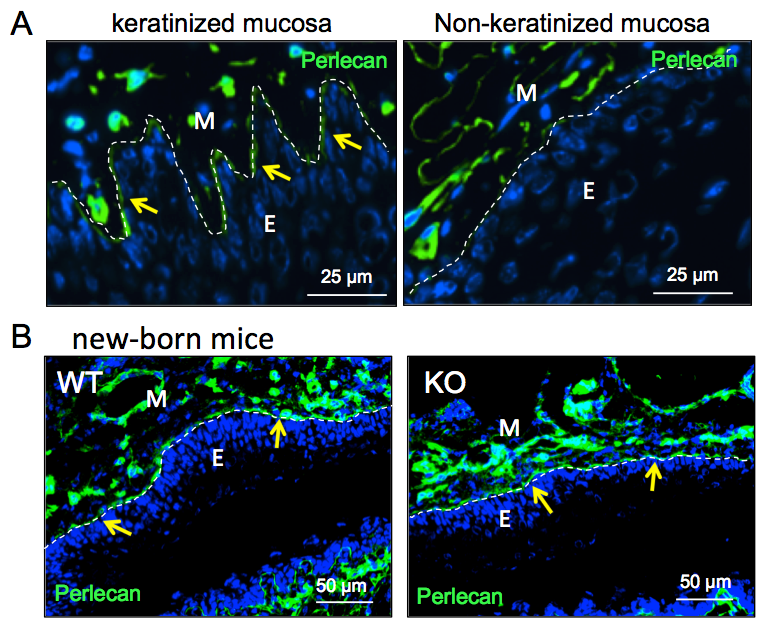


**Supplemental Figure 4**

hGECs were seeded in the ThinCert cell culture inserts (3D culture). Total RNA was collected after 0, 1, 3 and 7 days of culture. mRNA expression levels of *KRT5* (A), *KRT13*(B), *KRT14* (C), *KRT15* (D) and *KRT16* (D) were measured by real time RT-PCR. The expression of each gene was normalized to that of *S29* ribosomal RNA. Bars represent the mean values and standard deviation (+/−SD) (n=3). **p<0.01, ***p<0.001 (ANOVA, Tukey multiple comparison test).

**
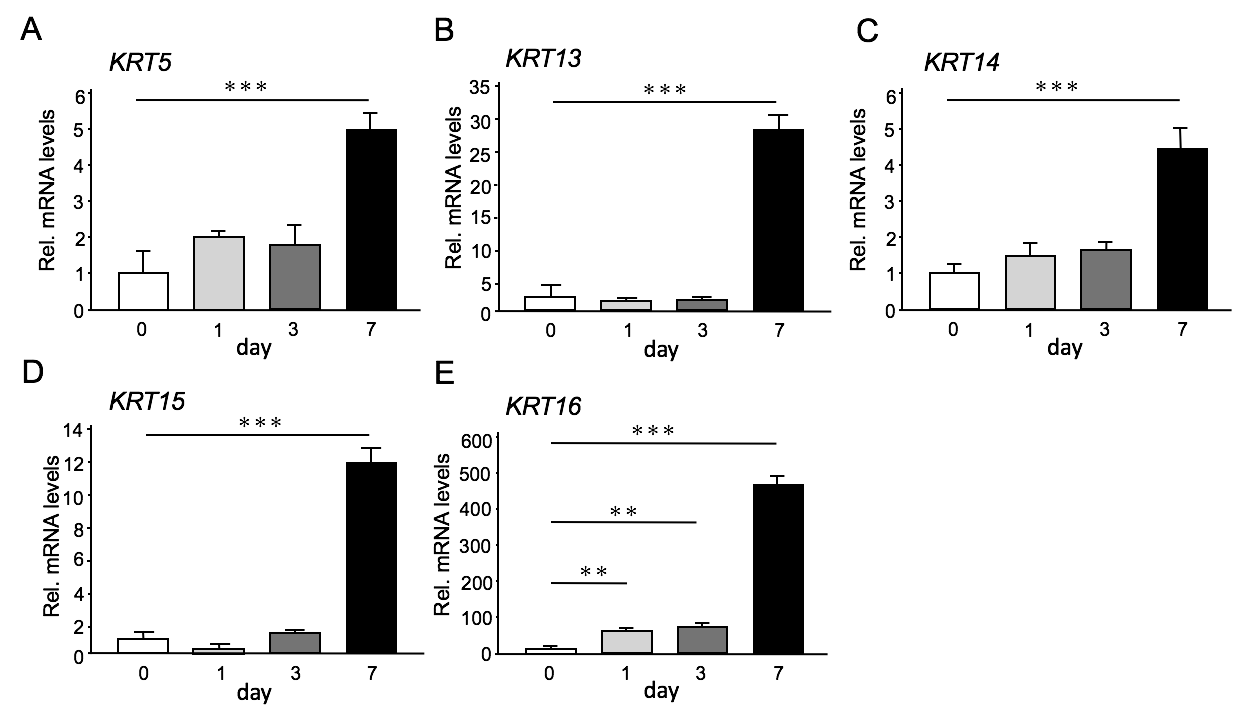
**

**Supplemental Figure 5**

hGECs were seeded in the normal culture plates (2D culture) and total RNA was collected at 1, 4, 8, 12, 20 and 28 days. mRNA expression level of *KRT10* was measured by real time RT-PCR. The expression of *KRT10* gene was normalized to that of *S29* ribosomal RNA. Bars represent the mean values and standard deviation (+/−SD) (n=3). (ANOVA, Tukey multiple comparison test).

**
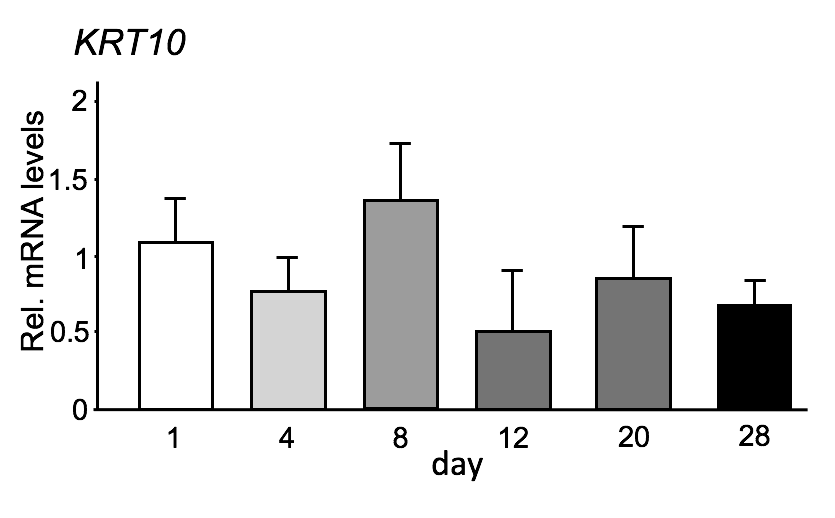
**

**Supplemental Figure 6**

hGECs transfected with siRNA targeting *COL4A6* gene were seeded in the ThinCert cell culture inserts, and total RNA and cell lysates were collected after 3 days. mRNA expression levels of *COL4A1* (A), *COL4A2* (B) and *COL4A5* (C) were measured by real time RT-PCR. The expression of each gene was normalized to that of *S29* ribosomal RNA. Bars represent the mean values and standard deviation (+/−SD) (n=3). ns: not significant (Student's t-tests). Results are representative of at least three independent experiments.

**
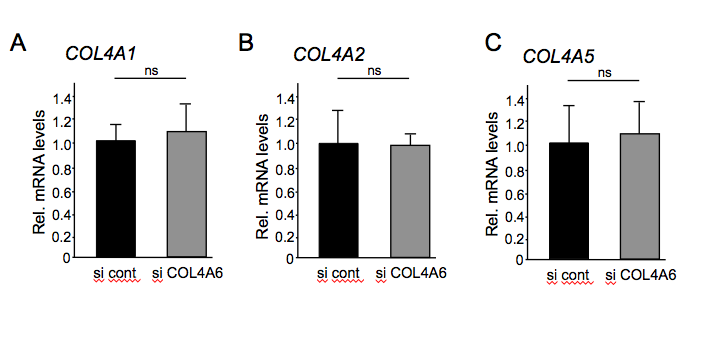
**

**Supplemental Figure 7**

Full length of western blot data shown in Figure 6D.

**
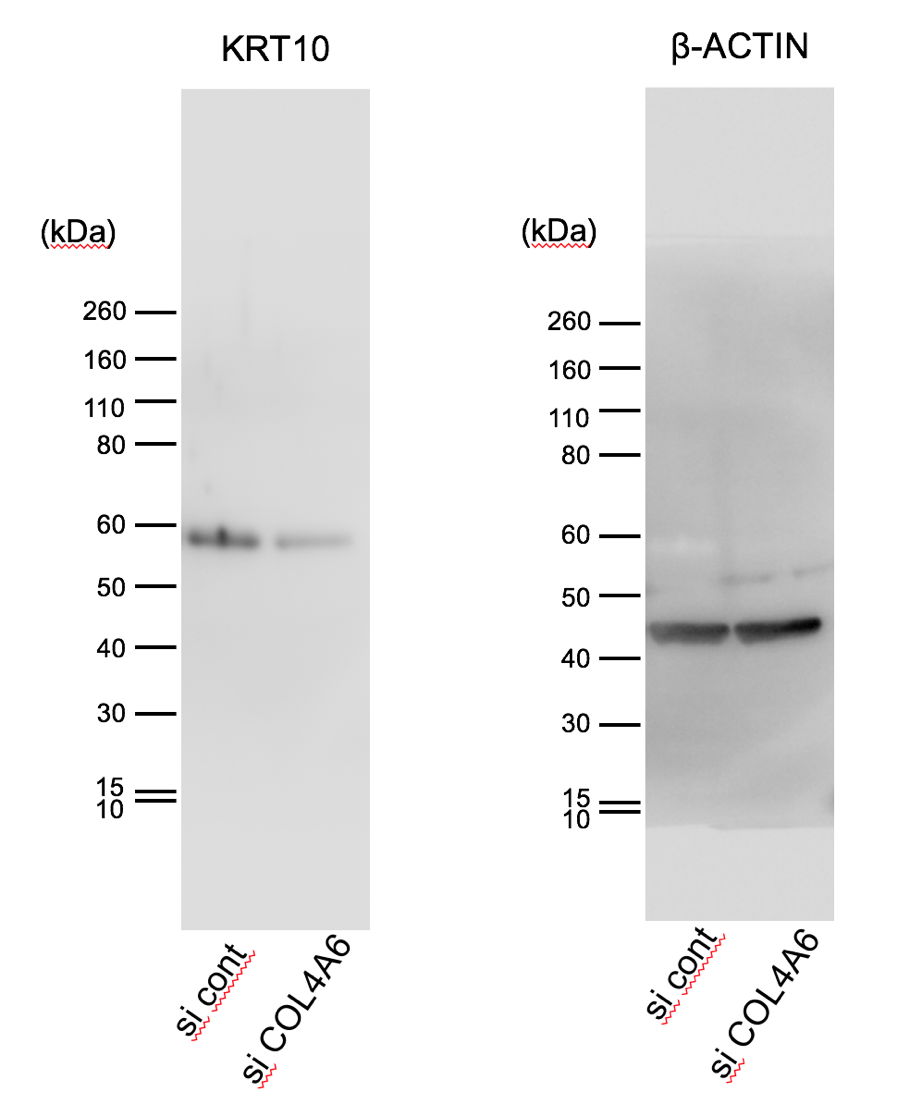
**
